# Novel Toll-like receptor-4 deficiency attenuates trastuzumab (Herceptin) induced cardiac injury in mice

**DOI:** 10.1186/1471-2261-11-62

**Published:** 2011-10-14

**Authors:** Nasser Ghaly Yousif, Fadhil G Al-amran

**Affiliations:** 1 University of Colorado Denver, Department of Medicine and Surgery 12700 E 19th Avenue, Aurora, CO 80045, USA; 2 Asadder Teaching Hospital, Al-najaf, Iraq; 3 Kufa University, Surgical Dept -College of Medicine, Najaf, Iraq

**Keywords:** Toll Like Receptor 4, cardiac-toxicity, Inflammation, trastuzumab

## Abstract

**Background:**

Cardiac inflammation and generation of oxidative stress are known to contribute to trastuzumab (herceptin) induced cardiac toxicity. Toll-like receptors (TLRs) are a part of the innate immune system and are involved in cardiac stress reactions. Since TLR4 might play a relevant role in cardiac inflammatory signaling, we investigated whether or not TLR4 is involved in trastuzumab induced cardiotoxicity.

**Methods:**

Seven days after a single injection of herceptin (2 mg/kg; i.p.), left ventricular pressure volume loops were measured in HeN compotent (TLR4^+/+^) and HeJ mutant (TLR4^-/-^) treated with trastuzumab and control mice. Immunofluorescent staining for monocyte infiltration and analyses of plasma by (ELISAs) for different chemokines including: MCP-1and tumor necrosis factor-α (TNF-α), Western immunoblotting assay for ICAM-1, and used troponin I for cardiac injury marker.

**Results:**

Trastuzumab injection resulted in an impairment of left ventricular function in TLR-4 competent (HeN), in contrast TLR4^-^/^- ^trastuzumab mice showed improved left ventricular function EF%, CO; p < 0.05, attenuation of mononuclear cell infiltration in TLR4 ^-/-^; p < 0.05 vs.TLR-4 competent (HeN), reduced level of cytokines TNF-α, MCP-1 and ICAM-1 expression in TLR4^-/-^, marked reduction of myocardial troponin-I levels in TLR4-deficient mice. Data are presented as means ± SE; n = 8 in each group p < 0.05 vs.TLR-4 competent (HeN).

**Conclusions:**

Treatment with trastuzumab induces an inflammatory response that contributes to myocardial tissue TLR4 mediates chemokine expression (TNF-α, MCP-1and ICAM-1), so in experimental animals TLR4 deficiency improves left ventricular function and attenuates pathophysiological key mechanisms in trastuzumab induced cardiomyopathy.

## Background

The human epidermal growth factor receptor (HER) proteins regulate cell growth, survival, adhesion, migration, and differentiation functions that are amplified or weakened in cancer cells. In some cancers, notably some breast cancers, human epidermal growth factor receptor-2 (HER2) is over-expressed, and, among other effects, causes breast cells to reproduce uncontrollably [1]. Trastuzumab is a humanized monoclonal antibody that binds selectively to the HER2 protein. and has become a mainstay in the treatment of women with (HER2) overexpressing breast cancer and in the metastatic and adjuvant settings this increases the survival of people with cancer [2]. One of the significant complications of trastuzumab is its effect on the heart and association with cardiac dysfunction in 2-7% of cases [3]. As a result, regular cardiac screening with either a MUGA (MUltiple Gated Acquisition) scan or echocardiography is commonly undertaken during the trastuzumab treatment period. Approximately 10% of patients are unable to tolerate this drug because of pre-existing heart problems; physicians are balancing the risk of recurrent cancer against the higher risk of death due to cardiac disease in this population. The risk of cardiomyopathy is increased when trastuzumab is combined with anthracycline chemotherapy (which itself is associated with cardiac toxicity) [4,5]

Toll-like receptors (TLRs) have a central role in innate immunity and inflammation, at least nine types of human TLRs have recently been identified [6] Among the family of TLRs, TLR4 has been the focus of particular interest since its recognition as a receptor for lipopolysaccharide (LPS; endotoxin) [7,8] It has been shown that active TLR4 led to expression of nuclear factor-κB (NF-κB)-controlled genes for proinflammatory cytokines that are required for activation of the immune response [9]

Previous study explained the Myocardial tissue TLR4 plays a major role in mediating myocardial injury following cold ischemia and reperfusion through up-regulation of MCP-1, (manuscript) [10]. Furthermore, increased TLR4 expression was observed in isolated cardiomyocytes from humans and animals with cardiomyopathies [11]. Growing evidence of a causal link between TLRs and the development of heart failure has been derived mostly from studies in knock-out mice supporting a relevant role of this receptor family. It had been shown that TLR4 can modulate LV hypertrophy, myocyte contractility, myocardial ischaemia reperfusion injury, and plays a role in inflammatory responses including septic shock syndrome [12]. It is notable that, cytokine release mediated by activation of the Toll- like receptors (TLRs ) is believed to be involved in the pathogenesis of doxorubicin induced cardiotoxicity [13,14] and are probably also involved in the development of doxorubicin induced cardiomyopathy, as has been shown in TLR2- deficient mice [15]. Identification of TLR4 ligands and elucidation of the mechanisms of ligand-TLR4 interaction may lead to the development of novel approaches for prevention of myocardial injury associated with trastuzumab treatment.

### Aim of the study

This study was done on mouse model to identify the effect of trastuzumab on TLR4 mutation in on the heart, leukocyte accumulation in the target area, MCP-1, ICAM-1, and the role of these chemokines in myocardial injury and leukocyte accumulation after treatment with trastuzumab.

## Methods

### Animals

Male C3H/HeJ mice (which have a point mutation in TLR4, resulting in a complete loss of signaling function) and C3H/HeN (wild-type) mice, body weight 24-30 g, acclimatized in a quarantine room for 2 weeks, and their age range from 8 to 12 weeks. All experiments were approved by the Animal Care and Research Committee of the University of Colorado Denver, and this investigation conforms to the Guide for the Care and Use of Laboratory Animals (National Research Council, revised 1996). The animals divided in to 4 groups, control groups injected with normal saline and other groups treated with 2 mg/kg trastuzumab in a single injection intraperitoneal i.p.

### Pressure-volume loop and hemodynamic analysis

Pressure-volume loop and hemodynamic analysis was only planned after 7 days of treated with trastuzumab, the mouse anesthetized with ketamin in dose of 50 mg/kg injected intraperitoneal and when the mouse anaesthetized, it will be positioned over the heating pad in supine position and four limbs are taped, in a orientation that the hind limb in front of operating researcher. Neck was opened longitudinally and right common carotid artery exposed and freed, ligated distally and stay suture placed proximally, then small opening was made in artery and size 1 F-micro tipped pressure transducer catheter (Millar Instruments, Houston, TX, USA) was inserted into the LV lumen via the right carotid artery for measurement of LV pressure, volume, function and related parameters. Then after about 20 minutes of data recording, the abdomen is opened by right sub costal incision to reach the inferior vena cava. To acutely change the cardiac preload, caval occlusion was produced over a 3-s period using a nonmetallic occluder applied to the IVC. The data were recorded as a series of pressure-volume loops.

P van software (Conductance Technologies, San Antonio, TX, and Millar, Houston, TX) was used to analyze all pressure-volume loop data. Regression analyses of multiple isochronal pressure-volume loop data were produced by IVC compression. From the baseline and IVC compression loops, comprehensive sets of hemodynamic parameters were calculated. All steady-state and caval occlusion pressure-volume loops were acquired with the computer data acquisition system. From these data we selected the following parameter: LV diameters were measured at end diastole (LVEDD) and end systole (LVESD), ejection fraction (EF), heart rate (HR), LV systolic pressure in the ends of both systole and diastole (LVESP, LVEDP), Cardiac Output (CO) and the maximum elasticity (Emax).

### Animal scarification

Immediately after finishing the pressure volume loop measurement, the mouse was sacrificed, starting by injection of equal volume of thiopental and heparin intraperotonealy in doses ranging from 100 μl to 200 μl for each one, after giving good time for the animal to go into deep anesthesia, the mouse is positioned and taped and the chest is opened in flap like manner revealing the heart then a needle of the syringe is introduced into right ventricle to aspirate around 0.5 ml of blood for plasma analysis. After that the heart is cut from the great vessels and mediastinum.

### Immunofluorescent Staining

Myocardial sections (5 μm thick) were fixed in 4% paraformaldehyde, incubated with a rabbit polyclonal antibody against PMNs and macrophages, and then incubated with Cy3-tagged secondary goat anti-rabbit IgG (imaged on the red channel). Nuclei were stained with bis-benzimide (DAPI, imaged on the blue channel), and glycoproteins on cell surfaces with Alexa 488-tagged wheat germ agglutinin (imaged on the green channel). Microscopy was performed with a Leica DMRXA digital microscope (Leica Mikroskopie und Systeme GmbH, Wetzlar, Germany). Immunoflourecent antibodies were used to target the macrophages and neutrophils.

### Plasma for protein assay

The collected blood from each mouse was centrifuged (in 10000 RPM, for 10 minutes at 4°C) and the yielded plasma samples of each animal was subjected to protein assay using enzyme-linked immunosorbent assays (ELISAs) for different chemokines including: MCP-1, tumor necrosis factor-α (TNF-α), and troponin I.

### Western immunoblotting assay

Myocardial tissue was homogenized with a rotor-stator homogenizer and treated in PBS containing 0.5% Triton X-100 and a protease inhibitor cocktail. Size fraction of crude protein (20 μg) was performed by electrophoresis. After transfer, the membrane was incubated in PBS 5% nonfat dry milk to block nonspecific binding. The membrane was then incubated for 60 minutes with an antibody against ICAM-1, or TLR4 at 1:1000 to 1:2000 dilutions with PBS containing 0.05% Tween 20 and 5% dry milk. After thorough washes, the membrane was treated with peroxidase-labeled secondary antibody (1:5000 dilutions with phosphate-buffered saline containing 0.05% Tween 20 and 5% dry milk) for 45 minutes. Protein bands were developed using enhanced chemiluminescence technique. Densitometry was performed using a computerized densitometer (Molecular Dynamics, Sunnyvale, CA).

### Statistical analysis

Statistical analysis was performed using the one-way ANOVA test with a significance of p < 0.05.

## Results

### TLR4 mutation improves LV function

Selected average parameters from pressure-volume loop study in the C3H/HeJ (TLR4^-^/^- ^) and C3H/HeN groups were shown in table-1, the C3H/HeJ (TLR4^-^/^-^) group has significantly better LV function and ventricular elasticity than the wild HeN group in form of EF%, and Cardiac Output (CO).

**Table 1:** Selected average parameters from pressure-volume loop study in the C3H/HeN and C3H/HeJ groups

Variables	HeN		HeJ		*P value
	Before Tras.	After Tras.	Before Tras.	After Tras.	
Heart rate (bpm)	449.67 ± 2.29	392.44 ± 16.47	452 ± 16	448 ± 31	<0.05
End-systolic Volume (uL)	13.42 ± 0.25	45.23 ± 0.42	14.04 ± 0.38	21.28 ± 0.60	<0.05
End-diastolic Volume(uL)	35.25 ± 0.36	63.77 ± 0.51	34.90 ± 0.55	41.40 ± 0.54	<0.05
End-systolic pressure(mmHg)	123.8 ± 0.47	179.35 ± 3.40	122.3 ± 1.03	139.22 ± 1.04	<0.05
End-diastolic pressure(mmHg)	8.2 ± 0.58	97.40 ± 3.53	7.97 ± 0.09	15.28 ± 0.17	<0.05
Ejection Fraction (%)	61.91 ± 8.9	29.06 ± 1.05	63.14 ± 2.17	52.17 ± 2.56	<0.05
Cardiac Output (ml/min)	10.44 ± 4.54	5.03 ± 0.57	11.01 ± 0.30	9.84 ± 0.28	<0.05

*Data are expressed as mean ± SEM; n = 8 in each group P value < 0.05.

### TLR4 ^-^/^- ^attenuated mononuclear cells infiltration

**Figure 1:**
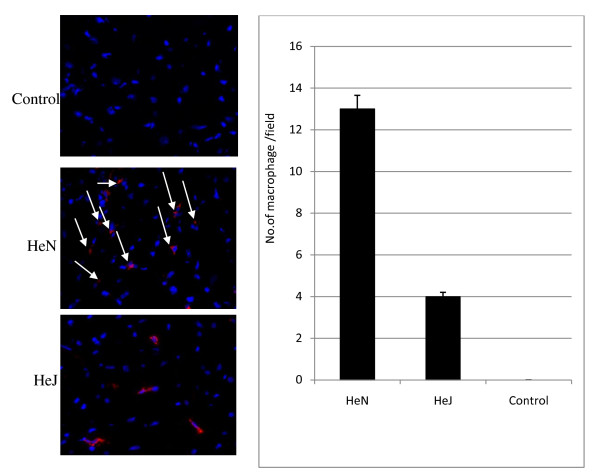
**Immunofluorescent Staining of the myocardial cells for demonstrated mononuclear cells infiltration**. Attenuated of infiltration mononuclear cells (white lines) in HeJ, data are presented as means ± SE; n = 8 in each group, p < 0.05 vs. TLR-4 competent (HeN)

After seven days of a single injection trastuzumab TLR4-competent (HeN-wild type) hearts exhibited marked increase in mononuclear cell infiltration compared with TLR4^-^/^- ^(HeJ mutant) hearts which is demonstrated by immunostaining as in Figure 1.

### Reduced level of cytokines TNF-a, MCP-1 and ICAM-1 expression in TLR4 ^-^/^-^

**Figure 2:**
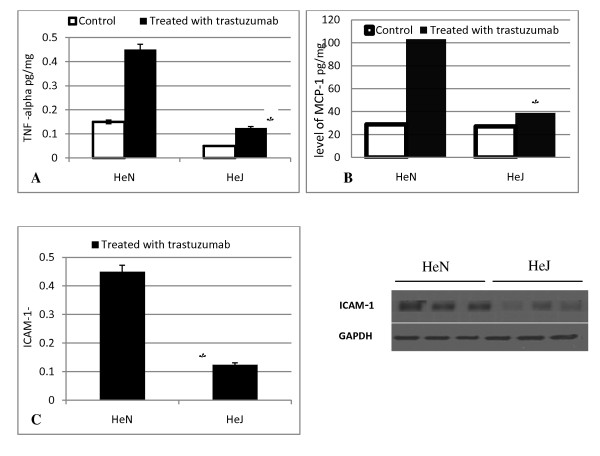
**level of cytokine (MCP-1, ICAM-1andTNF-α) in myocardial tissue and serum**. Blood samples and hearts isolated from C3H/HeN (TLR4 competent) and C3H/HeJ (TLR4 defective) mice were subjected to single injection of trastuzumab (2 mg/Kg i.p). While control hearts were subjecting to normal saline i.p. (A) level of TNF-α (B) level of monocyte chemoattractant protein-1 (MCP-1).(C) ICAM-1 was obtained after harvest heart, Western immunoblotting assay was performed as described in Materials and Methods, markedly reduced ICAM-1 in the He J mutant (TLR4^-^/^-^).

To characterize the cardiac inflammatory response due to the trastuzumab injection, we determined cardiac cytokines, expression of TNF-α protein, MCP-1and ICAM-1. led to an increased expression both of them in WT(C3H/HeN) mice compared to TLR4 ^-/- ^(HeJ mutant) (*P *< 0.05) Figure 2.

### Marked reduction of myocardial troponin-I levels in TLR4-deficient mice

**Figure 3:**
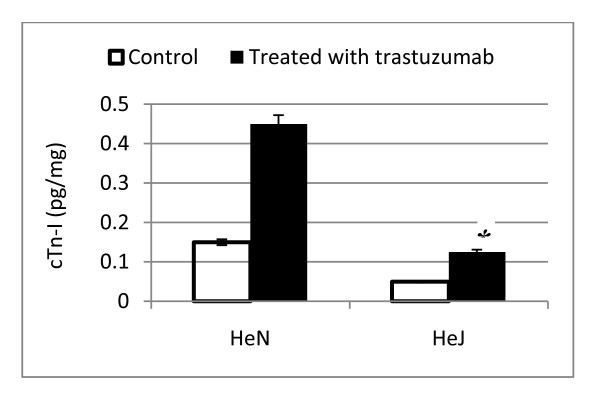
**Level of the serum cTn-I in the mice group**. Reduced serum levels cTn-I in the HeJ mutant (TLR4^-/-^) group. Data are expressed as means ± SE; *n *= 8 in each group; P < 0.05 vs. HeN treated with trastuzumab.

The level of the troponin-I as cardiac injury marker show significant reduction in C3H/HeJ strains after seven days a single dose injection of trastuzumab, and this reflected that the cardiac injury is more in wild type, as shown in Figure 3.

## Discussion

The antitumor functions of trastuzumab are associated with its ability to modulate signaling through the HER-2/*neu *receptor as well as initiate antibody-dependent cell-mediated cytotoxicity (ADCC) [15]. Recent studies suggest that trastuzumab disrupts HER2/HER3 interactions, leading to downregulation of AKT signaling, which results in decreased cell proliferation [16-18]. At any stage, the use of trastuzumab reduces recurrence by about 50% and increases overall survival by about 30% [19-21] and reduction in the risk for death by about 44% (*P *< .0001, hazard ratio [HR] = 0.56) [22].

In approximately 4% of patients with trastuzumab therapy are associated with severe congestive heart failure, and most of the cardiac toxicity seen with treatment is limited to asymptomatic decreases in the left ventricular ejection fraction (LVEF); however [23].

In the present study, we tested the hypotheses that TLR4 mediates mononuclear infiltration and chemokine expression that response to underlying trastuzumab treatment. We found that TLR4-competent mice treated with trastuzumab display severe systolic and diastolic LV dysfunction, resulting in impaired cardiac output as measured by the assessment of pressure - volume loops, while in TLR4^-^/^- ^cardiac output was improved as a result of enhanced systolic and diastolic performance, and reduced mononuclear infiltration with significantly lower levels of TNF-α, MCP-1 and ICAM-1. These result of correspond to myocardium injury after global ischemia and reperfusion led to TNF- expression and other cytokines [24], and is under the control by myocardial TLR4 [25], TNF- may serve as an intermediate in TLR4-mediated myocardial, including IL-8 and MCP-1. In myocardiac regional ischemia and reperfusion (I/R) demonstrate a critical role of TLR4 in myocardial chemokines response [26].

The John Cha [27] and his group studies show that the TLR4 signaling is involved in the myocardial inflammatory response after global ischemia/reperfusion and that TLR4 signaling contributes to cardiac dysfunction after global ischemia/reperfusion through its influence on myocardial production of TNF-α and IL-1 peptides. The Kaczorowski and colleagues [28] in which their data demonstrate that TLR4 signaling is central to both the systemic and intragraft inflammatory responses that occur after cold I/R in the setting of organ transplantation.

In our earlier study we found that the Myocardial injury after global cold ischemia and perfusion was evaluated by serum cardiac troponin-I (cTn-I) as cardiac marker injury that was decreased levels in HeJ, p < 0.05 vs. TLR4 competent (HeN) [29,30] also we found the same sequel in the present study the serum levels of cTn-I in the HeJ mutant (TLR4^-/-^) was reduced.

According to our knowlage no study was done that link between the TLR4 and trastuzumab treatment, so our data demonstrate for the first time a relevant role of TLR4 in the development myocardial injury following used trastuzumab under experimental conditions. Further studies using therapeutic interventions such as pharmacological TLR4 inhibition are required.

## Conclusions

This study demonstrates that treated with trastuzumab induces an inflammatory response that contributes to myocardial tissue TLR4 as mediates chemokine expression (TNF-α, MCP-1and ICAM-1), so in experimental animals TLR4 deficiency improves left ventricular function and attenuates pathophysiological key mechanisms in herciptin-induced cardiomyopathy.

## Competing interests

The authors declare that they have no competing interests.

## Authors' contributions

NGY carried out the plasma for protein assay, Western immunoblotting assay, participated in the design of the study and performed the statistical analysis, participated in the sequence alignment and drafted the manuscript. FGA carried out the Pressure-volume loop and hemodynamic analysis and participated in the design of the study. All authors read and approved the final manuscript.

## Pre-publication history

The pre-publication history for this paper can be accessed here:

http://www.biomedcentral.com/1471-2261/11/62/prepub
